# Access to mental health and psychosocial services in Cambodia by survivors of trafficking and exploitation: a qualitative study

**DOI:** 10.1186/s13033-015-0008-8

**Published:** 2015-03-18

**Authors:** Charlotte Aberdein, Cathy Zimmerman

**Affiliations:** London School of Hygiene & Tropical Medicine, London, UK; Department of Global Health & Development, London School of Hygiene & Tropical Medicine, London, UK

**Keywords:** Mental health services, Psychosocial services, Availability, Accessibility, Abuse, Exploited, Trafficked

## Abstract

**Background:**

Emerging evidence indicates the extreme forms of violence and acute and longer-term mental health consequences associated with trafficking and exploitation. However, there has been little research on post-trafficking mental health and psychosocial support services for survivors. This study explored the availability and accessibility of mental health and psychosocial support services in Cambodia for women, men and children trafficked and exploited for sex or labour purposes.

**Methods:**

Semi-structured interviews were conducted with a purposively selected sample of representatives from seven service organizations providing mental health and psychosocial support services for people who have been trafficked. This qualitative method was selected to gain insights into the service approaches and challenges faced by the small number of post-trafficking service providers in Cambodia. A conceptual framework outlining access dimensions associated with service provision guided the structure of the study.

**Results:**

Findings indicate that among the available post-trafficking services, there are few trained mental health specialists, an over-representation of shelter services in urban versus rural areas and limited services for males, people with disabilities, individuals exploited for labour (versus sexual exploitation) and those with more serious mental illnesses. Providers believe that discrimination and stigma related to both mental health and human trafficking hinder trafficked people’s willingness to access services, but suggest that awareness-raising may reduce these prejudices. Care in this sector is precarious due to over-reliance on financial support by donors versus government. Recent increases in newly qualified professionals and providers suggest potential improvements in the quality and availability of psychological support for trafficking survivors.

**Conclusions:**

Psychological support for the growing number of identified trafficking survivors in Cambodia will depend on improved geographical distribution of services, more mental health support professionals and growing acceptability of mental health service use among trafficked people and the Cambodian public.

## Background

Cambodia is renowned for its history of genocide, decades of conflict, foreign occupation and political oppression. The loss of approximately one third of the population under the Khmer Rouge (1975–79) and mass bombing campaigns contributed to well-documented prevalence of traumatic stress among Cambodian survivors [1-3]. Today, Cambodia, like other countries in the Mekong region, is also known for the trafficking of human beings. Human trafficking has been defined in the UN Protocol to Prevent, Suppress and Punish Trafficking in Persons as the recruitment, transportation, transfer, harbouring or receipt of persons, by means of the threat or use of force or other forms of coercion, of abduction, of fraud, of deception, of the abuse of power or of a position of vulnerability or of the giving or receiving of payments or benefits to achieve the consent of a person having control over another person, for the purpose of exploitation [4].

The covert nature of human trafficking and problems in defining who is considered ‘trafficked’ hinder prevalence studies globally and within Cambodia [5]. Statistics are unreliable with reported declines in the number of victims identified in some instances whilst also acknowledging that victim identification procedures are poor and true figures are likely to be much higher [6]. For example, official data shows that despite large numbers of women and girls trafficked to, from and within Cambodia for forced sex work [5-7], the number of referrals to NGOs by government has declined from 388 in 2013 to 151 in 2014 [6]. Meanwhile, one organisation reported repatriating twice as many Cambodian male victims of trafficking from Mauritius, Indonesia, Senegal, Malaysia, and South Africa in 2013 than in 2012 [6].

However, a recent survey with a random sample of 2690 adults from nine provinces conducted by the Royal University of Phnom Penh found trafficking more prevalent in Cambodia compared to previous data from Asia. In 0.7% of the households surveyed one to four members of the family had experienced trafficking; 61.1% in the previous year [8].

Cambodian women and girls are trafficked for domestic work (especially within the region, e.g., Malaysia) [9,10], while men and boys are commonly trafficked for industrial fishing, and Cambodian children are trafficked for begging and prostitution. Throughout this cycle of debt-bondage and exploitation, trafficked people are frequently subjected to extreme forms of abuse and deprivation [9].

These multiple exposures to violence can result in acute and longer-term mental health problems. Recently, a small body of evidence has provided some indication of the mental health outcomes experienced by trafficked women [11-14]. Only one study has looked at health outcomes of men and women who had been trafficked for labour exploitation in the UK [15].

The past and current violence in Cambodia has taken its toll on the mental health of both the older and younger Khmer generations. Yet to date, the mental health needs of Cambodians have remained largely unaddressed, partly due to the almost complete destruction of the health system under the Khmer Rouge. Mental health services were not available until psychiatry received some attention following elections in 1993 and gradually services have been reintroduced [16,17].

## Prevalence of mental illness in Cambodia

Mental health is defined by the WHO as ‘a state of wellbeing in which every individual realizes his or her own potential, can cope with the normal stresses of life, can work productively and fruitfully, and is able to make a contribution to her or his community’ [18].

Available data on the prevalence of mental illness among Cambodians are limited with the majority of research being conducted with Cambodian refugees in the United States. No data exists on the prevalence of mental health problems associated with human trafficking and exploitation in Cambodia. However, data that exists for the general population suggest a higher percentage of mental illnesses than normative populations [1-3]. Through a structured literature review, two studies were identified indicating the prevalence of psychiatric disorders among the general population in Cambodia [2] and war-affected Cambodians living in Cambodia [1].

Dubois et al. examined the association between psychiatric symptoms, including post-traumatic stress disorder (PTSD), depression and anxiety, with an impaired social function among Cambodian adults in Kampong Cham province [2]. Of the study population, 7.3% met criteria for PTSD and 53% displayed symptoms associated with anxiety disorders. The study found increased risk for social impairment among respondents displaying signs of co-morbidity (depression, anxiety and PTSD).

A second study conducted by De Jong et al. [1] explored the association between common mental disorders (PTSD, mood disorder, somatoform disorder, and anxiety disorders) and exposure to violence [2]. Findings indicated that among respondents exposed to violence, symptoms of anxiety were most prevalent, affecting 42.3% (38 · 9–46 · 7) of the sample, with 33.4% (29 · 2–37 · 6) measuring positive for symptoms of PTSD [1].

Mental disorders have been strongly associated with violence, rapid social change, insecurity and trauma [19,20]. Further, findings from various studies in low-income countries (LICs) suggest that mental health and poverty can interact in a negative cycle, potentially exacerbating one another [19,20]. It is worth noting that the latest Demographic and Health Survey (DHS) figures for Cambodia indicate that approximately 28% of the Cambodian people live below the poverty line [21].

## Developing mental health services

Over the past decade, there has been a rapid rise in the number of post-trafficking services run by non-governmental organisations (NGOs) [16]. This service proliferation may be an indication of both the increasing numbers of exploited individuals requiring assistance, as well as the rising donor attention to the problem. In an internationally comparative public health analysis of sex trafficking of women and girls in eight cities the researchers identified a major gap in all eight cities of culturally sensitive mental health services [22].

At the same time as post-trafficking service provision increases in Cambodia, many challenges emerge related to the development of mental health services in general and for supporting trafficking survivors, in particular. General challenges include, for example, a shortage of skilled human resources, limited financial resources, poor collaboration between service providers and an unequal distribution of the limited professional mental health support that is available [23]. Currently, there are only 49 trained psychiatrists in the country, of whom only 10 operate outside of the capital Phnom Penh in addition to 45 psychiatric nurses who operate across the whole country in mental health facilities and/or private practices [8,24,25,]. Service delivery challenges are exacerbated by a lack of leadership and investment in mental health, reflected by the meagre 1% of a US$2.6 billion health budget for 2012 allocated to mental health [26]. Further to this, of the international donors who contribute to Cambodia’s health budget few have shown an interest in funding the mental health sector [25]. Similar service challenges are common in low and middle income countries (LMICs) [27]. Moreover the invisible and intransigent nature of mental health problems can further hinder a dedicated and robust response among policy makers and service providers [27].

Nonetheless, in Cambodia, mental health services have seen some notable improvements in recent years [23]. Figures for 2012 indicate that 300 doctors and as many nurses completed basic mental healthcare training, and that medical students at the University of Health Sciences are now able to specialise in psychiatry [26]. Also, in 2012, the first 22 students graduated with a Bachelor of Arts in Social Work from the Royal University of Phnom Penh (RUPP) [28]. RUPP is the leading institution for mental health training in Cambodia, having run a bachelor’s degree programme for psychology since 1994, and now offering a 2-year master’s in Clinical Psychology and Counselling. Since the course was established in 2008, this master’s programme is currently training a third cohort of students, with 19 due to complete their studies in 2014. Figures available for 2012 indicate that 660 students have graduated with a bachelor’s degree in psychology and 13 students had graduated with a master’s degree, with many working in private practices as programme coordinators and counsellors in the fields of trauma and mental health [8]. The lead researcher contacted the Head of Psychology Department at RUPP, Ms Sek Sisokhom, who confirmed that to date all graduates of the master’s in Clinical Psychology and Counselling had gone on to work in NGOs.

There is limited evidence on Cambodian services for trafficked people. However, findings from one study on Cambodia’s capacity to assist trafficked persons suggest that while there is a growing network of service providers that facilitate knowledge and skills transfer [16] serious questions persist about the competency, qualifications and oversight of staff assisting trafficked persons [16].

In Cambodia, mental health support may come under either the rubric of *mental health* or *psychosocial support,* which are often used interchangeably. However, the literature on mental health and psychosocial responses has attempted to differentiate the terms in the following way:

“Mental health tends to use labels related to psychopathology and connote illness and disease… Psychosocial problems are often situational and relational and are within range of what is considered normal behaviour under abnormal situations” [16].

In Cambodia, “psychosocial services” provided to trafficked people consist of meeting their immediate needs, often through crisis-stage care which can include shelter, rehabilitation and reintegration. As noted by Devine psychosocial approaches do “not deny or exclude the need for more psychological and/or psychiatric interventions by specialists” [16].

## Evidence on the psychological effects of trafficking and exploitation

Oram et al. conducted a systematic review of research on trafficking identifying 19 studies, all of which included trafficked women and girls only and focused primarily on trafficking for sexual exploitation [29]. Four studies described mental health outcomes [12-14,30]; two studies focussed on people trafficked for labour exploitation [12,13] and none looked at males. The studies included in this review found high levels of anxiety (48.0%-97.7%), depression (54.9%-100%) and PTSD (19.5%-77.0%). Hossain et al. found a trafficking experience of 6 months or more could be linked to increased levels of mental distress (OR: 2.23 CI: 1.10-4.53) [30].

A new study by Turner-Moss et al. [15] looked at a sample of 35 men and women, of which three quarters were male (77%), who had been trafficked for labour exploitation in the UK. The study found that eighty-one percent (25/31) reported one or more physical health symptom and fifty-seven percent (17/30) reported one or more post-traumatic stress symptom [15].

Abas et al. [31] have carried out the first study to describe risk factors for diagnosed mental disorders in female survivors of human trafficking. The analysis, a historical cohort study of women aged 18 and over who returned to Moldova, found childhood sexual abuse and post-trafficking stressors (poor social support and greater unmet needs) were independent risk factors for mental disorder [31].

Robust evidence from Cambodia is limited but one qualitative study focused on the experiences of trafficked girls, and concluded that mental distress is common among girls, who described depression, PTSD-like symptoms, and mood and anxiety disorders [32].

### Available treatments

In Cambodia two main trauma-focused interventions are being used to process the memory of trauma and/or its meaning; trauma-focused cognitive behavioural therapy (TF-CBT), a structured psychological intervention, has been used successfully by a number of organisations with girls who were trafficked for sex [32,33]. TF-CBT is a psychotherapeutic model designed for youth, that integrates elements of cognitive behavioural, humanistic, attachment, family, and empowerment therapies to respond to trauma [34]. A number of students and psychologists have also been trained in eye movement desensitization and reprocessing (EMDR) which has been shown to be as efficacious as TF-CBT [8,35]. EMDR is an information processing therapy that helps to alleviate PTSD symptoms enabling people to process the meaning of the event through integration of memories into their autobiographical memory [35].

Cognitive behavioural approaches have strong empirical support [36] but rely on the availability of skilled psychiatrists trained in their use. TF-CBT has been adopted to work with sexually abused children due to its proven efficacy in other settings [37,38] and there is consistent evidence showing that trauma-focused treatments result in considerably larger effects than non-trauma-focused interventions [33].

Professionals in the field advocate for the effectiveness of psychosocial alternatives, but measuring efficacy proves more difficult.

This study explores access to MHPSS in Cambodia for trafficked and exploited people.

## Methods

To research service availability and use, we drew on the concept of *access*, as conceived by Penchansky and Thomas [39] who theorised that ‘access’ had multiple dimensions, including: availability, accessibility, accommodation of client needs, affordability and acceptability [39].

Purposive sampling was used to identify participants from among the limited number of service providers offering post-trafficking care. This study utilises expert sampling to obtain particular knowledge and expertise. The sample was selected based on the lead author’s personal knowledge of post-trafficking services in Cambodia and through conversations with experts working in the field of anti-trafficking and mental health. Ten organisations were invited to participate and seven agree to take part in the final study.

The organisations participating in this study broadly fall into two groups. The first group are often referred to as ‘post-trafficking services’. They provide services to survivors of sex and labour trafficking but also respond to other forms of exploitation common in Cambodia, including sexual exploitation and rape, domestic violence, child abuse and acid attacks (organisations 1, 5, 6, and 7). The second group of organisations in this study provide mental health, psychosocial or social services to the wider population, alongside those who have been trafficked. (organisations 2, 3 and 4).

In addition to the MHPSS outlined in Table 1 below, all of these organisations provide the following, to a greater or lesser extent, depending on their priorities and expertise: recovery shelters, legal support, education and employment programmes, healthcare, trauma counselling and transitional and reintegration support.

**Table 1:** **Characteristics of participants and organisations**

**NGO**	**Operational**	**Overview of services provided**	**Primary target population**	**Number of therapists**	**Professional role of participants**	**Professional background**
Organisation 1	National	Social work & support services for male survivors of sexual abuse & their families	Boys & men but also work with families	8 social workers and counsellors	Technical Advisor	Social Work
Organisation 2	National	Community-based mental health services & training in mental health/social services	General	8-10 social workers and 1 social work coordinator	Director/Advisor	Social Work
Organisation 3	National	Community mental health programmes	General	3 psychiatrists, 5 psychologists, 4 counsellors, 1 psychiatric nurse, 1 social worker	Project Coordinator – Rehabilitation of Victims of Human Trafficking	Psychology
Organisation 4	Regional	Therapy or treatment of trauma, specialists in EMDR	General	Information not available	Psychologist	Clinical Psychology
Organisation 5	International	Provision of social workers trained in crisis care to people affected by sex trafficking & CSE	Girls – no successful interventions involving boys to date	Information not available	Director of Aftercare (post-trafficking care)	Social Work
Organisation 6	International	Recovery, shelters, use of TF-CBT as a trauma therapy	Women, children, work with men through families	15 counsellors, separate team of case managers and social workers	Psychosocial Advisor	Counselling, Therapy
Organisation 7	International	Operates short-term Assessment Centre for female clients	Girls, 6-17	Information not available	Counsellors (2)	Counselling

Financial information available for five of the organisations indicates that they are funded through a range of international donors. Figures for 2013 indicated that organisations 4 and 5 received the highest proportion of funds from individual contributions; 88% and 72% respectively. Foundations were the most significant funding source for organisation 6 (42%) and provided the second highest source of income for organisation 5, followed by funds from organisations’ partners, churches, and governments. Organisation 6 received the highest proportion of government funding (10%). They also received 8% from corporations and 2% from enterprise partners.

A descriptive overview of data about the clients of the seven participating organisations is presented in the results. The type of data available varies by organisation, partly due to different monitoring and data collection processes but also due to confidentiality. To supplement the data provided by the participants, additional data was obtained from annual reports and program evaluations.

Semi-structured interviews were conducted via Skype with seven mental health professionals in Cambodia, who were working in the fields of social work, psychology, clinical psychology and counselling. In total there were eight participants from seven organisations; two counsellors participated from organisation 7 (see Table 1). Four participants were Khmer and four were expatriates who primarily held director or advisor roles. Two were male and six female. Where participants were not fluent in English, a translator was present. Interview guides were structured around the access framework and included semi-structured questions that allowed participants to describe their perceptions, while also enabling the researcher to compare their responses.

The questions were designed to enable the participants to present their own opinions and experiences in addition to those of their organisation. For instance, in relation to ‘accessibility’ participants were asked - “are you aware of any mental health services being accessed by those still being held by their trafficker? – whereas other questions were asked to acquire organisation-specific information – “does your organisation charge fees to the client for mental health services?”.

The interview guide consisted of 30 open-ended questions. This method was chosen to learn the opinion, beliefs, experience and reported behaviour of the participants without prescribing response options or leading participants to a particular answer. The interview was divided into three sections and began with a background question about the participants education, training and current role, followed by three questions to elicit information about who their organisations’ services were geared towards, what mental health needs they encountered, and how their organisation responded to the specific mental health needs of trafficked and exploited people. The second section posed questions to shed light on broader structural and political challenges to providing MHPSS with a specific question on the policy context and implications. The participants were also asked their perspective on the challenges to providing “effective mental health interventions for this population” and also the level to which they were involved in “delivering an effective mental health intervention for this population”, and whether it was scaled-up. The third section was structured around the five ‘access’ dimensions; availability, accessibility, accommodation, affordability and acceptability. For each dimension several questions were asked to explore a wide range of access issues. The questions to explore ‘affordability’, for instance, covered whether the organisations charged fees for services; if financial assistance following a referral improved access; the existence of health social assistance schemes; and whether there are any benefits to the client of providers charging small fees.

All interviews were audio recorded on both a handheld digital recorder and on computer software and transcribed by the researcher. Participants were offered information on the ‘access’ framework prior to the interview. Interviews were analysed using standard social science methods for analysing qualitative data. Interview transcriptions were inductively coded, line-by-line using NVivo software 9.2 to identify repeatedly emerging themes and categorise data. The initial index codes were drawn from the main question themes, with further emerging codes identified (e.g. monitoring and evaluation, lay worker training etc.). Text associated with each main code could then be analysed, and additional sub-codes developed. The coding resulted in 13 main codes and 32 sub-codes (words and phrases used to tag sections of the transcripts). For example, some of the sub-codes under ‘acceptability’ are cultural appropriateness, type and location of facility, and religious affiliation. Coding the transcript enabled the researcher to assign values of frequency, presence and absence, and relationship with other codes. Data were then synthesised to foster cross-cutting findings.

Electronic databases, Medline, Global Health and PsychINFO, were searched using keywords and subject headings. ELDIS was used to search grey literature, supplemented by a comprehensive search on the public domain for additional grey literature and reports. The references of key papers were checked for additional papers missed in the main search and expert recommendations were also sought. Due to the limited evidence on the mental health consequences of trafficking and exploitation it was necessary to include non-peer reviewed literature.

This research was approved by the Ethics Committee at the London School of Hygiene and Tropical Medicine. All participants in the study were assured of confidentiality and anonymity using written agreements. All identifiers have been removed to protect participant confidentiality.

## Results

### Client and therapist characteristics^a^

Data on the proportion of male and female clients between 2011 and 2014 is available for three of the organisations; organisation 4 has data for 2011–2013, organisation 1 for 2014, and organisation 6 for 2013.

Organisation 1 provides services for approximately 100 clients each year, of which approximately 80 per cent are male and 20 per cent female. Although this organisation’s target beneficiaries are primarily male, services also extend to the client’s male and female family members.

Organisation 4 provided services for 1,483 clients between 2011 and 2013; 1137 of these were adults (739 female and 368 male), 346 were children (169 female and 177 male) and 30 were transsexual adult clients.

Data for the total number of clients served is also available for organisations 2 and 6. Organisation 6 provided services to 677 clients in 2013; 25 received TF-CBT. Between 2009 and 2011, organisation 2 provided services to 207 clients; 80% of these received services in their community and 20% at the shelter.

Age-disaggregated data is only available for organisation 2. 18% (37/207) of clients were under 10 years of age, with the highest number of clients in the 10–18 year-old age group (35%). 84% of all the clients were under the age of 30.

Rape was the most common reason for referrals of clients served by organisation 2 (140/207 cases), followed by attempted rape, gang rape, sexual harassment, forced marriage and trafficking. Data for 2011 shows that organisation 3 provided counselling services to 54 cases of trafficking (17 females and 37 males) and psychiatric treatment to five clients suffering from severe psychiatric problems.

#### Availability

Challenges to the availability of MHPSS reported by participants included unequal geographical distribution of services, the low priority placed on MHPSS by some organisations, narrow targeting to specific populations, such as those referred into shelter-based models of care (i.e., aftercare). One participant in an advisory role for a community-based service organisation explained:

*We are trying to influence the way the system works and move away from shelter-based services being the only model. It means unless you go to a shelter you cannot access services. Unless someone is in danger of being re-trafficked or is in an incest situation we don’t see any reason why someone has to be away from home and it actually slows down their recovery.*

The importance of varying models of support tailored to differing individual needs are noted, such as services for survivors who do not need or want to reside in a shelter setting, but still wish to access support.

Significant service gaps in the provision of MHPSS were identified for three groups in particular: male survivors; labour exploitation survivors (more organisations address sex trafficking or child exploitation); and people affected by more serious mental illnesses. A Technical Advisor working specifically with male survivors of sexual abuse described the service challenges in assisting males:

*Often boys will be blamed, isolated, marginalised and punished for expressing the problems they have. A boy might act out, be aggressive and anti-social. This is a particular barrier to providing services and there is a real need to understand the perspective of men and what help they need.*

A psychosocial advisor emphasised the neglect of people with more serious mental illnesses, such as schizophrenia, bi-polar, severe depression and severe anxiety disorders:

*I think they [people with serious mental illnesses] go largely undiagnosed and there is not a lot of capacity for working with them… there needs to be a short-term place where people can live and be supervised and have therapy every day but there is nothing like that here at all.*

These providers emphasise the low priority placed on responding to the needs of these marginalised groups who can be even more vulnerable to abuse and exploitation.

Community or provincial level services were reportedly very limited, although organisations are beginning to pay greater attention to this geographical gap, as one psychosocial advisor explained the move towards providing more community-based services rather than bringing people into shelters.

Two participants, including a counsellor and director of aftercare, highlighted the inadequate and limited training opportunities for staff. Scarce opportunities for wider training of support persons means there are only a handful of practicing Khmer specialists. This provider training gap was noted by two counsellors working in a short-term assessment centre for trafficked women and girls:

*Not many people have the opportunity for specialist training here in Cambodia. Most people have on-the-job training and attend short courses.*

One participant [director of an aftercare program] underlined the importance of on-going professional training:

*There is a strong handful of staff that are well trained but I would still consider them to be in non-specialist roles. They can provide effective interventions but need continuing training, input, supervision and support.*

Overall participants agreed that the necessary human resources are scarce. One psychosocial advisor explained:

*There are not nearly enough psychiatrists and qualified counsellors; although there are many people doing psychology degrees, they have not had enough experience of working with clients.*

While there is progress as more students graduate from psychology and social work programmes, a participant [aftercare director] stated that “even that education only goes so far”, again suggesting the importance of on-going, on-the-job training.

Referring to the high numbers of Vietnamese women and girls often encountered in cases of exploitation, one advisor for community-based mental health services indicated the importance of having Vietnamese-speaking staff and noted that this is an “important part of the next stage of [service] development”.

A staff member from one organisation estimated that 15 per cent of clients from their sexual assault programme were disabled, and the staff found it extremely difficult to find supported accommodation for these individuals once their programme ended. The staff member also emphasised that children with disabilities are an extremely neglected client-group, who are often especially vulnerable to abuse, explaining:

*Children with disabilities are considered very low value and often do not have the capacity to see or tell who their perpetrator is.*

#### Accessibility

Participants explained that post-trafficking services are often accessed based on a referral by social workers or the police and rarely sought by themselves. The technical advisor working with males explained that after time, their service began receiving self-referrals via word-of-mouth, though some people have difficulties phoning an NGO.

*Some clients have accessed us through other clients so here it is word-of-mouth. This is really effective. Doing outreach in communities is key.*

This provider emphasised the importance of careful consideration of barriers specific to the population that services are trying to reach.

Societal blame and stigma is a common reason for people’s reluctance to seek out services. As one advisor noted “societal blame is particularly strong for victims of sexual exploitation and trafficking”. Disclosure may damage both the individual’s and the family’s reputation.

Among general community-based mental health services, several are beginning to employ various mechanisms to raise awareness about their services among the wider population. Three participants [psychologist, advisor, project coordinator] described the use of radio programmes, stickers, announcements in newspapers, and leaflet distribution. On the other hand, post-trafficking services mainly rely on referrals to provide information to clients, due to confidentiality and security concerns.

The disparity between urban and rural area service availability was raised by all participants. A technical advisor working with male survivors of exploitation explained:

*One of the greatest disappointments of the way things have developed in Cambodia is that because services are very much centre-based there are very few services in the community. We deliberately didn’t build another drop-in centre.*

Yet, outreach to the rural areas appears to be an area that is receiving emerging attention and action. One participant [psychosocial advisor for post-trafficking service] explained that they plan to start providing satellite teams in the provinces.

At the same time, this participant also recognised the advantage for clients of staying in post-trafficking care facilities (e.g. shelters or aftercare):

*All our services are available to them, they don’t have to seek it out, and it is just given as part of our services. I think for others it is very difficult for them to get access.*

The advantages of delivering services in the community were also identified by an advisor supporting those delivering community-based MHPSS:

*People tend to feel flattered by being visited at home. Often in cases where family members are upset, it gives us a chance to work with the families as well.*

This participant highlights a more holistic approach that includes the family, suggesting that in some cases this may contribute to longer-term recovery, depending on the nature of the family relationship.

The absence of post-trafficking services at the community-level was also identified as a challenge to reintegration, as noted by a psychosocial advisor working with trafficked women:

*When trafficked women return from Malaysia they are very keen to return to their village…They are desperate to get back to their communities but when they get there they don’t have access to services.*

Again, this suggests an urgent need to find ways to make services readily available in the provincial areas for those who want continued support, who also prefer to regain some independence and control by returning to a ‘normal’ routine (i.e., not living in a shelter).

To improve access to care, all participants explained that their organisations provide varying levels of assistance with transport and financial support. One organisation working with males reimburses their clients for lost earnings for the time taken off work to access services.

*For people earning $3 a day that is a huge amount of money to lose and becomes a barrier to access.*

To date, this type of compensation for income loss to access services appears to be more of an exception than common practice.

#### Accommodation of client needs

Participants were asked questions about how well they, as a provider were able to accommodate the needs of the client, (e.g., confidentiality, hours of operation, etc.). The importance of safe physical and emotional environments to foster a sense of security and trust was underlined by all participants. The technical advisor for one organisation noted:

*It is about helping them to create safe spaces in relationships and us helping them to identify what their needs are.*

Client-centred care is emphasised, especially during this early stage of rehabilitation. One [technical advisor] commented:

*We try to deliver client-centred social work which is all about the client’s needs, not the needs of the organisation.*

For example, a psychologist providing therapy for treatment of trauma expressed the preference for telephone counselling services among individuals responding to radio announcements advertising their services. This participant explained that while they were not specifically trained in telephone counselling, they wanted to be as responsive as possible to people’s needs.

Residential services may also offer more flexible hours for support and assistance, as they operate around the clock. However, several staff working for organisations with outpatient facilities or providing community-based services noted that they do their best to meet with clients at evenings and weekends.

#### Affordability

Nearly all services offered by providers who were interviewed are free of charge; however, sustaining free services depends on continued funding. One project coordinator for rehabilitations of trafficked people confirmed:

*For trafficked people we provide services free of charge. Consultation fees and medication fees are covered for the duration of the project.*

Participants agreed that charging for services will deter health-seeking behaviour, especially for mental health, where there are already so many other access challenges. The technical advisor for services for men and boys explained that they have a client assistance fund that supports additional expenses to help clients with, for example, accommodation and training.

Health and social assistance schemes are also limited, and, as one advisor noted, concerns about confidentiality might prevent people from enrolling in public support schemes.

#### Acceptability

Ensuring that services were ‘acceptable’ to clients was a goal repeated by each participant. One psychosocial advisor suggested that they have staff who can meet the diversity of their clients’ needs:

*We have male and female staff, Cambodian and Vietnamese, married, single, older, younger, Christian and Buddhist. If the clients are not happy they can change counsellors.*

Although all providers said they try to take age, gender, ethnicity and religion into consideration, they also noted that matching a client’s needs often depends on the capacity of available staff.

Two participants, [technical advisor and advisor/director] specifically recognised the ways some clients draw strength from their religious or spiritual beliefs during recovery and their role in supporting this.

*I remember we had one girl in the shelter who wanted to go to church and others who would go to the wat [Buddhist temple].*

However, a third participant also expressed concern about situations where religious perceptions about certain types of services might compromise an individual’s access to specialist care:

*There is an issue with some faith-based NGOs, if they reject social work. Whilst I respect their beliefs, if that means children are denied access to much-needed specialist help, this worries me.*

Questions about how to assess the capacity and suitability of referral organisations was also raised by a number of participants. For example, one aftercare director specifically expressed concern about organisations that might make participation in religious activities a criterion for service eligibility:

*We ask about their religious affiliation and what religious activities look like on a practical level, also what happens if a client chooses not to engage and how our clients are offered the opportunity to engage or not.*

Cultural relevance was mentioned by all participants. For example, culturally appropriate psychometric tools were described by one participant, who referred to the assessment tool: CEPAT,^b^ which had been designed specifically for the Cambodian context by researchers at John Hopkins University [40]. A psychologist providing treatment for trauma to the general population, including some trafficked people, emphasised the importance of adapting and validating tools and methods prior to using them in Cambodia:

*We translate our screening tools and use them in Khmer [local language]; we try not to purely translate but keep the meaning and make it relevant. Sometimes this is still a problem because we don’t have lots of ‘feeling’ words in Khmer.*

The limitations of Western concepts to describe psychological symptoms or mental health support in the Cambodian context were also mentioned by another participant:

*I remember one boy who said, ‘I don’t know what counselling is but if someone can listen to my feelings and listen to me talk, then that would be good’.*

As noted, despite Cambodia’s extraordinary population exposure to traumatic events, there is very limited history of mental health service provision, thus many people are still unfamiliar with the purpose and methods of counselling.

Reducing stigma and discrimination was noted as an important way to improve service acceptability. Four participants explained the benefits of educating staff and, where possible, the wider population, about the effects of abuse in order to foster a better understanding and acceptance of their clients. One provider explained:

*We conduct training that is compulsory for everyone including our drivers and administrative staff so that everyone understands the needs of a person who has survived; I think this is reducing the stigma.*

It was suggested that including both clinical and non-clinical staff (e.g., administrative staff) in anti-discrimination training will help reduce pre-conceptions and discriminatory behaviour that may affect client uptake of services.

#### Other challenges to the availability of MHPSS

Providers were offered the opportunity to raise additional service challenges not necessarily covered within the access framework.

Several participants noted the lack of available financial resources, particularly within the non-governmental organisations and the ministries, such as the Ministry of Social Affairs, Veteran, and Youth Rehabilitation (MOSAVY), responsible for providing support to trafficked people.

Additionally, participants stated that poor collaboration between stakeholders posed many challenges. Dissension between services was highlighted as particularly problematic by an advisor for one community-based service:

*It is really hard for donors to come in and help them because some people won’t talk to each other or attend the same meetings.*

One project coordinator for a mental health program explained that when clients come to them as a health professional, clients frequently expect pharmaceutical medication for symptoms, even for symptoms that may be treatable through counselling alone.

Although Trauma-Focussed Cognitive Behavioural Therapy has been used in Cambodia, this treatment is certainly not favoured by all mental health professionals. As one participant [technical advisor], expressing doubt about whether this method is appropriate, suggested the need for caution when considering this form of treatment:

*TF-CBT is cheap, it’s measurable and actually is the opposite of what social work stands for which is looking deeply into many of those other issues.*

There remain conflicting opinions about the effectiveness of TF-CBT as a treatment for survivors of trafficking and exploitation.

When discussing the government’s commitment to improving the availability of MHPSS, all participants agreed that there was very weak commitment. Corruption, poor understanding of trafficking and mental health and the low priority placed on health were identified as considerable barriers to prioritising rigorous responses. However, one participant providing services for male survivors added that some government officials seem eager to learn about the issues:

*We have recently had discussions with Excellencies from the Ministry of Women’s Affairs and they are desperate to learn, they are very embracing of these issues.*

## Discussion

The following is an assessment and interpretation of the results supported by evidence. Overall our results are consistent with existing literature on access to MHPSS in LMICs. Our findings indicate that access depends significantly on the geographical distribution of services and the ability of services to support the needs of under-served and marginalised groups (e.g., men, disabled, sexual minorities). Care is also related to the scarcity of professional psychological support persons and the limited training options for existing post-trafficking support staff. Furthermore, service provision for trafficking survivors in Cambodia is often narrowly focussed on those who need or are willing to enter residential shelter models, with fewer service options for those who choose to reside outside a refuge.

There were several limitations to this research that may hinder the generalisability of the results. Most significantly, the findings from this study are drawn from a small number of professionals working in civil society and are therefore not representative of all services providing MHPSS; government or private. Using a semi-structured approach with open-ended questions meant that at times the detail was not relevant to the research or difficult to analyse. However, the interviews provided rich data, with great insight into access to MHPSS from a range of perspectives, and allowed participants to raise issues that the researchers had not considered. The use of Skype technology, without the video function, at times, allowed the participant to talk freely and with more confidence because they could not be seen.

Researcher selection bias was also a limitation, due to the purposive nature of the sampling technique. Whilst this limits the external validity of the findings, other sampling methods, such as probability sampling, would only have been feasible if there were a greater number of eligible non-government organisations providing services to this population. Notably, other qualitative studies about mental health in Cambodia, with similar numbers of participants had comparable results, supporting these findings. These findings also complement earlier literature that highlights the scarcity of resources to support the mental health of survivors, the over-centralisation of services in urban versus rural areas; and that inefficiencies in service delivery [41], often related to staff capacity and limited opportunities for professional education and training, can leave survivors without well-implemented mental health support [30,42]. Findings are also reinforced by the consistency across the interviews.

Of the three organisations unable to participate in the study, one was established specifically to respond to sex trafficking through prevention, rescue, restoration and reintegration. The second is a large international NGO working on a range of issues in development and humanitarian contexts, including trafficking and exploitation with a regional programme focusing on prevention, protection and policy. The third organisation provides Creative Arts Therapy to organisations working with trafficked and abused children.

If the research looked more broadly at access to MHPSS by the whole population it would have been critical to include government and private provider perspectives. However, the purpose of this research was to explore the perspective of mental health professionals providing services to trafficked and exploited people. Currently, government authorities refer survivors of trafficking and exploitation to NGO providers; since 2013 MOSAVY have referred 310 cases of trafficking to NGOs [6].

According to one source, over the last couple of years the number of ‘reported’ sex trafficking cases has declined while the number of identified victims of labour trafficking has increased [6]. While the nature of trafficking may be changing there is a consistent need for organisations providing MHPSS to broaden their remit to adapt and strengthen their capacity. Furthermore, to address geographical inequalities in service availability, providers may have to offer incentives for trained professionals to relocate to the provinces, and institute professional mental health or psychosocial training for community-based service staff in the rural areas. In the past, services for trafficking survivors focussed on support for women trafficked for sexual exploitation, thus it is not surprising that current services are not yet prepared to meet the needs of males and those exploited for labour purposes. A 2012 report stated that more than 100 male Cambodian victims were repatriated from Thailand after being trafficked for forced labour on Thai-flagged fishing boats [9]. Moreover, services appear unprepared to support individuals affected by more serious disorders and people with disabilities. To a certain extent, providers may improve their capacity to serve these populations by strengthening their coordination and inter-sectoral referral network, for example, between post-trafficking care, general MHPSS and services for marginalised populations (e.g. those with disabilities) to bridge gaps.

Yet, at the same time, until public and professional discrimination and stigmatisation of trafficked people and people suffering mental health problems can be reduced, individuals in need of support may continue to avoid seeking help for fear of increasing their marginalisation. Educating professionals may be easier than sensitising the public, but until the public attitude is more understanding and accepting, trafficking and psychological morbidity may remain hidden and/or stigmatising problems.

At present, there did not appear to be a wide range of well-recognised mental health intervention approaches. Moreover, there were somewhat conflicting views about the use of TF-CBT in this setting. Although there is some evidence indicating the effectiveness of TF-CBT among similar populations [36], several professionals advocated a less structured, more holistic approach to treatment. Intervention trial-style research may help answer questions about the best approach, and for example, whether TF-CBT might have the most effective results if used as part of a holistic response to mental health and psychosocial needs.

Importantly, Cambodian culture is a lens through which people view their psychosocial wellbeing and treatment expectations. Our findings emphasise the importance of culturally acceptable services, especially where there is limited knowledge of Western concepts for MHPSS. So-called culture-bound syndromes within Cambodians have been explained by use of cultural narratives and idioms [42], for example, a “weak heart” resembles a mix of PTSD and panic disorder [43]. Considerable effort is being made to adapt and validate diagnostic and screening tools, which may strengthen diagnostic validity [44]. Nonetheless, the translation process remains a challenge because the Khmer language often lacks words with the equivalent meanings to describe emotions and symptomatology.

Clients’ preference for medication for physical expressions of psychological problems seems to pose a challenge to providers, who were concerned about over-medicating mental illness - and this finding coincides with previous research that clients’ requests for medication were a particular problem for government services [26]. The preference for or reliance on medication may reflect both Cambodian’s unfamiliarity with counselling methods and the extremely limited availability of counselling services. It is possible that NGOs might play an important role in raising awareness about the range of post-trauma reactions, particularly the physical expression of stress and distress and non-pharmaceutical forms of treatment.

Importantly, there appear to be a promising number of new professional degree programmes that may foster an increasing number of mental health providers. However, it remains unclear whether these professionals will be able to find work providing post-trafficking counselling and whether they will be motivated to move to the provinces, where there is the greatest shortage of mental health support. At the same time as there might be a growing body of professionals, there remains a need for more formal training and support for individuals working with survivors of trafficking and exploitation by Cambodian trained professionals. To date, many NGOs rely on expatriate specialists for treatment or training, which is not a sustainable model. At this point in time, organisations are utilising the resources available to provide what is often-ad-hoc and on-the-job training to staff. Formal training for staff and supervision of service delivery is necessary.

Because many people who have been trafficked may not wish to enter residential services, in the future, it will be important for Cambodian services to be designed as ‘out-patient’ or community-based MHPSS. In the case of individuals who face serious security risks, residential programs may be essential, but for others, residing away from their family and support network this may ultimately hinder their recovery. Additionally, residential care may increase an individual’s risk of unwanted disclosure of their trafficking experience or identification as an individual seeking mental health support, exacerbating both actual and perceived stigma associated with both.

Reintegration has been identified as a particularly important aspect of the post-trafficking recovery process [12], yet previous research on post-trafficking care found very weak support systems for the reintegration of trafficked persons [16]. Community-based MHPSS may be most well positioned to foster effective and sustainable reintegration by providing service options closer to an individual’s home, and without the confinement of institutionalisation. Similarly, donors and service organisations may wish to consider the benefits of telephone counselling services, which may further reduce the risk of unwanted disclosure and overcome some of the geographical barriers to service access.

Whether a trafficked person uses a MHPSS is highly dependent on whether they are aware that this type of service exists and have the information about how to access this service [17]. Our findings show that information often reaches survivors via referrals (police, immigration) or sometimes when information is distributed to the community. In the future, providers may have to improve the means by which they communicate the availability of their services, such as through radio, news and other media networks, where appropriate. Word of mouth may also become an effective means of communication, provided that survivors are engaged in awareness strategies.

While there appear to be government services available, official and unofficial fees for government health services remain a challenge to service use [45]. Similarly, individuals may be opting not to use health social assistance schemes for fear of stigma, and breaches of confidentiality. The provision of free and accessible care is also dependent on sustained funding from donors, but mental health has suffered from a low position on international and national agendas [24,27].

The intrinsic values of the organisation can critically influence a provider’s perception of acceptability through service provision. Our results suggest that a spiritual factor can be important in an individual’s recovery, but some expressed concern in cases where religious values negate the perceived necessity for the provision of specialist care.

In addition to the practical and logistical problems with services, our research reinforced the view that weak government commitment and corruption is limiting national response to the mental health needs of trafficked, abused and exploited people. Moreover, because government commitment is lacking, these services are only sustainable with international donor support.

## Conclusions

This research has highlighted factors that can influence access to MHPSS for trafficked and exploited people and suggested potential strategies to improve services. While positive steps are being taken by a number of service providers to decentralise services and develop community-based MHPSS, these initial actions must be a catalyst for farther-reaching change, especially in the professional and public attitude towards particularly vulnerable groups, such as trafficked persons and people experiencing mental health problems.

## Endnotes

^a^ Refer to Table 1 for corresponding organisations.

^b^ Child Exploitation Psychosocial Assessment Tool.

## Abbreviations

CEPAT: Child exploitation psychosocial assessment tool
CSE: commercial sexual exploitation
DHS: Demographic and Health Survey
LICs: Low-income countries
LMICs: Low- and middle-income countries
MHPSS: Mental health services and psychosocial support services
MoH: Ministry of Health
MOSAVY: Ministry of Social Affairs, Veteran, and Youth Rehabilitation
NGOs: Non-governmental organisations
PTSD: Post-traumatic stress disorder

## References

[1] de Jong J, Komproe I, Van Ommeren M (2003). Common mental disorders in postconflict settings. Lancet.

[2] Dubois V, Tonglet R, Hoyois P, Sunbaunat K, Roussaux JP (2004). Household survey of psychiatric morbidity in Cambodia. Int J Soc Psychiatry.

[3] Sonis J, Gibson J, de Jong J, Field N, Hean S, Komproe I (2009). Probable posttraumatic stress disorder. JAMA.

[4] United Nations Office of Drugs and Crime. United Nations Convention against Transnational Organized Crime and the Protocols Thereto. 2004. http://www.unodc.org/documents/treaties/UNTOC/Publications/TOC%20Convention/TOCebook-e.pdf. Accessed 23 Jul 2012

[5] Haworth A. Virginity for sale: inside Cambodia’s shocking trade. In: The Observer. 2014. http://www.theguardian.com/society/2014/jul/06/virginity-for-sale-cambodia-sex-trade. Accessed 06 Jul 2014.

[6] U.S. Department of State. 2014 Trafficking in Persons Report. 2014. http://www.state.gov/j/tip/rls/tiprpt/countries/2014/226693.htm. Accessed 3 Jan 2015.

[7] McCauley HL, Decker MR, Silverman JG (2010). Trafficking experiences and violence victimization of sex-trafficked young women in Cambodia. Int J Gynaecol Obstet.

[8] Schunert T, Khann S, Kao S, Pot C, Saupe LB, Lahar C, et al. Cambodian Mental Health Survey - Royal University of Phnom Penh, Dept of Psychology. 2012. http://www.giz.de/Entwicklungsdienst/de/downloads/Cambodian_Mental_Health_Survey_Online_final_12.6.14.pdf. Accessed 28 Dec 2014.

[9] U.S. Department of State: 2012 Trafficking in Persons Report. http://www.state.gov/j/tip/rls/tiprpt/2012/192366.htm. Accessed 23 Jul 2012.

[10] Norodom S. Human trafficking must be stopped. In: The Phnom Penh Post. 2012. http://www.phnompenhpost.com/columns/human-trafficking-must-be-stopped. Accessed 22 Jun 2012.

[11] Zimmerman C, Hossain M, Watts C (2011). Human trafficking and health: a conceptual model to inform policy, intervention and research. Soc Sci Med.

[12] Ostrovschi NV, Prince M, Zimmerman C, Hotineanu MA, Gorceag LT, Gorceag VI (2011). Women in post-trafficking services in Moldova: diagnostic interviews over two time periods to assess returning women’s mental health. BMC Public Health.

[13] Tsutsumi A, Izutsu T, Poudyal AK, Kato S, Marui E (2008). Mental health of female survivors of human trafficking in Nepal. Soc Sci Med.

[14] Cwikel J, Chudakov B, Paikin M, Agmon K (2004). Trafficked female sex workers awaiting deportation: comparison with brothel workers. Arch Womens Ment Health.

[15] Turner-Moss E, Zimmerman C, Howard LM, Oram S (2014). Labour exploitation and health: a case series of Men and women seeking post-trafficking services. J Immigr Minor Health.

[16] Devine S. Psychosocial and Mental Health Service Provision for Survivors of Trafficking. Baseline Research in the Greater Mekong Subregion and Indonesia. International Organization for Migration. 2009. http://publications.iom.int/bookstore/free/PsychosocialandMentalHealth%28Eng%29.pdf. Accessed May 17 2012.

[17] Somasundaram DJ, Van de Put W, Eisenbruch M, de Jong JT (1999). Starting mental health services in Cambodia. Soc Sci Med.

[18] WHO. Mental health: a state of well-being. 2014. http://www.who.int/features/factfiles/mental_health/en/. Accessed 30 Dec 2014.

[19] Patel V, Kleinman A (2003). Poverty and common mental disorders in developing countries. Bull World Health Organ.

[20] Lund C, Breen A, Flisher AJ, Kakuma R, Corrigall J, Joska JA (2010). Poverty and common mental disorders in low and middle income countries: a systematic review. Soc Sci Med.

[21] Hor D, Tung R, Loun M, Hong R, Barrère B, Fishel J, et al. Cambodia Demographic and Health Survey 2010. In: Measure DHS. 2011. http://dhsprogram.com/pubs/pdf/FR249/FR249.pdf.

[22] Macias Konstantopoulos W, Ahn R, Alpert EJ, Cafferty E, McGahan A, Williams TP (2013). An international comparative public health analysis of sex trafficking of women and girls in eight cities: achieving a more effective health sector response. J Urban Health.

[23] Stewart J (2011). Mental health in Cambodia: a qualitative evaluation international organization for migration.

[24] Mental Health Crisis Strains Cambodia. In: Voice of America. 2010. http://www.voanews.com/content/mental-health-crisis-strains-cambodia-90925709/165591.html. Accessed 31 Aug 2012.

[25] McLaughlin D, Wickeri E. Special Report: Mental Health and Human Rights in Cambodia. Leitner Center for International Law and Justice. 2012. http://www.leitnercenter.org/files/2012%20Leitner%20Cambodia%20Report%20(with%20photos).pdf. Accessed 23 Feb 2013.

[26] IRIN. Humanitarian News and Analysis. What ails Cambodia’s mental health system? 2012. http://www.irinnews.org/report/95054/. Accessed 20 Jun 2012.

[27] Saraceno B, van Ommeren M, Batniji R, Cohen A, Gureje O, Mahoney J (2007). Barriers to improvement of mental health services in low-income and middle-income countries. Lancet.

[28] Yang C. Cambodia’s first social work grads ready to take the reins,” *Phnom Penh Post*. 2012. http://www.phnompenhpost.com/lifestyle/cambodia’s-first-social-work-grads-ready-take-reins. Accessed 17 May 2012.

[29] Oram S, Stöckl H, Busza J, Howard LM, Zimmerman C. Prevalence and risk of violence and the physical, mental, and sexual health problems associated with human trafficking: systematic review. PLoS Med. 2012;9 doi:10.1371/journal.pmed.1001224. 10.1371/journal.pmed.1001224 PMC3362635 22666182

[30] Hossain M, Zimmerman C, Abas M, Light M, Watts C (2010). The relationship of trauma to mental disorders among trafficked and sexually exploited girls and women. Am J Public Health.

[31] Abas M, Ostrovschi NV, Prince M, Gorceag VI, Trigub C, Oram S (2013). Risk factors for mental disorders in women survivors of human trafficking: a historical cohort study. BMC Psychiatry.

[32] Bolton P, Nadleman S, Wallace T. Qualitative Assessment of Trafficked Girls in Cambodia. John Hopkins University. Boston University. World Vision. 2008. http://www.childrecovery.info/fileadmin/pdf/Qualitative_Assessment_Cambodia_2008.pdf. Accessed 8 Jun 2012.

[33] Ehring T, Welboren R, Morina N, Wicherts JM, Freitag J, Emmelkamp PMG (2014). Meta-analysis of psychological treatments for posttraumatic stress disorder in adult survivors of childhood abuse. Clin Psychol Rev.

[34] Bass J, Bearup L, Bolton P, Murray L, Skavenski S. Implementing Trauma Focused Cognitive Behavioral Therapy (TF-CBT) among Formerly Trafficked-Sexually Exploited and Sexually Abused Girls in Cambodia : A Feasibility Study. 2011. http://www.childrecovery.info/fileadmin/pdf/TF-CBT_Feasibility_Report_Cambodia_2011.pdf. Accessed 13 Jun 2012.

[35] McGuire T, Lee C, Drummond P (2014). Potential of eye movement desensitization and reprocessing therapy in the treatment of post-traumatic stress disorder. Psychol Res Behav Manag.

[36] Lalor K, McElvaney R (2010). Child sexual abuse, links to later sexual exploitation/high-risk sexual behavior, and prevention/treatment programs. Trauma Violence Abuse.

[37] Ehlers E, Clark DM, Hackmann A, McManus F, Fennell M (2005). Cognitive therapy for post-traumatic stress disorder: development and evaluation. Behav Res Ther.

[38] Cohen JA, Deblinger E, Mannarino AP (2004). Trauma focused cognitive behavioural therapy reduces PTSD more effectively than child centred therapy in children who have been sexually abused. J Am Acad Child Adolesc Psychiatry.

[39] Penchansky R, Thomas W (1981). The concept of access: definition and relationship to consumer satisfaction. Med Care.

[40] Khun, C. Assessing the psychosocial needs of trafficked and sexually exploited girls using the Child Exploitation Psychosocial Assessment Tool (CEPAT). 2011. http://www.childrecovery.info/Child-Exploitation-Psychosocial-Assessment-Tool.166.0.html. Accessed May 23 2012.

[41] Saxena S, Thornicroft G, Knapp M, Whiteford H (2007). Resources for mental health: scarcity, inequity, and inefficiency. Lancet.

[42] Rechtman R (2000). Stories of trauma and idioms of distress: from cultural narratives to clinical assessment. Transcult Psychiatry.

[43] Hinton D, Hinton S (2010). The Khmer ‘weak heart’ syndrome: fear of death from palpitations. NIH Public Access.

[44] Ommeren M (2003). Validity issues in transcultural epidemiology. Br J Psychiatry.

[45] Bigdeli M, Ir P. A role for user charges? Thoughts from health financing reforms in Cambodia. In: Health systems financing - the path to universal coverage. The World Health Report 2010. Background Paper, 42. World Health Organization. Accessed 10 Jan 2015. http://www.who.int/healthsystems/topics/financing/healthreport/UF_HEF_No42.pdf.

